# A Systematic Study of Rotation Robustness in Remote Sensing Image Segmentation: RICM Versus Random Rotation Augmentation

**DOI:** 10.3390/s26175674

**Published:** 2026-09-07

**Authors:** Zhanpeng Huang, Haihui Wang, Yuhang Wang, Longbin Yu, Xinzhi Cao

**Affiliations:** 1 School of Computer Science and Engineering, Wuhan Institute of Technology, Wuhan 430205, China; forever0139@163.com (Z.H.); 22407010152@stu.wit.edu.cn (Y.W.); 22407010084@stu.wit.edu.cn (L.Y.); 22407010122@stu.wit.edu.cn (X.C.); 2 Hubei Key Laboratory of Intelligent Robot, Wuhan Institute of Technology, Wuhan 430205, China

**Keywords:** remote sensing image segmentation, rotation robustness, random rotation augmentation, rotation-invariant learning, Rotation-Invariant Channel Mapping (RICM), semantic segmentation, U-Net

## Abstract

This study presents a systematic comparison of two dominant paradigms for improving rotation robustness in remote sensing semantic segmentation: rotation-invariant architectural modules (represented by Rotation-Invariant Channel Mapping, RICM) and random rotation data augmentation. Despite both approaches being widely adopted, a fair and side-by-side evaluation under unified experimental conditions remains lacking. Using U-Net as the baseline on the LoveDA dataset, we comprehensively evaluate six strategies: RICM alone, random rotation augmentation at probabilities p=0.25 and p=0.5, and their combinations. Performance is assessed under both discrete rotations (0°, 90°, 180°, 270°) and continuous angles (0°–180° with 15° intervals), supplemented by Rotation Consistency (RC) analysis, class-wise evaluation, and computational cost comparison. Cross-dataset validation on ISPRS Potsdam confirms generalizability. Our results demonstrate that: (1) random rotation augmentation consistently outperforms RICM, delivering substantial robustness gains with zero inference overhead; (2) augmentation probability controls a clear accuracy–robustness trade-off, with p=0.25 achieving the best balance (maintaining 0° mIoU at 74.8% while boosting 90° mIoU from 54.9% to 63.1%) and p=0.5 achieving near-invariance at the cost of reduced baseline accuracy; and (3) RICM offers only marginal benefits and becomes redundant when augmentation is applied. Analysis of the observed performance patterns suggests that early-stage RICM insertion may suppress useful orientation-specific features and that its rotation-ensemble averaging may be insufficient for dense prediction tasks. Overall, random rotation augmentation proves to be a simple, effective, and computationally efficient strategy for improving rotation robustness in remote sensing segmentation.

## 1. Introduction

### 1.1. Research Background and Motivation

Semantic segmentation of remote sensing imagery is a fundamental task supporting diverse applications such as urban planning, disaster response, land-cover mapping, and precision agriculture [1]. In recent years, deep convolutional neural networks (CNNs), especially U-Net [2] and its variants, have achieved remarkable success in this domain. However, unlike natural images that are generally captured with an implicit upright orientation prior (e.g., ImageNet [3]), remote sensing images exhibit a distinctive property: objects may appear at arbitrary orientations due to varying sensor viewpoints, flight trajectories, and scene layouts. Consequently, the same semantic category—roads, buildings, or vehicles—can present drastically different orientations across images.

Although convolution operations are inherently translation-equivariant, they are not rotation-equivariant [4]. As a result, even minor rotations of the input can provoke substantial changes in feature responses, leading to unstable and often erroneous segmentation outputs. Three categories of rotation-induced errors are particularly consequential for remote sensing applications. First, **road network fragmentation**: roads are strongly directional structures, and a model trained on horizontally or vertically aligned roads may misclassify rotated road segments as building boundaries or background, producing broken road connectivity that directly undermines transportation mapping and network analysis. Second, **building boundary distortion**: buildings in urban scenes typically exhibit orthogonal layouts, and rotated buildings may lose their regular rectangular shape in the prediction, leading to inaccurate footprint boundaries that compromise urban planning, area estimation, and change detection. Third, **small object omission**: thin or small objects such as vehicles, narrow roads, and utility structures are especially vulnerable; rotation changes their effective aspect ratio in the feature space, and the model may fail to detect them entirely—a critical failure in disaster damage assessment and traffic monitoring. These errors are not minor: as our experiments demonstrate, a conventional U-Net can suffer a nearly 20-percentage-point mIoU drop when the input is rotated by 90°, rendering segmentation outputs unreliable for most practical remote sensing workflows.

Since remote sensing images of the same geographic area are frequently acquired from different angles, such rotation sensitivity critically undermines the reliability of downstream analyses. Note that “original orientation” throughout this paper refers to the orientation in which images appear in the training distribution (typically north-up for orthorectified remote sensing products), not an absolute standard. Remote sensing imagery has no canonical orientation, but models trained on a consistent orientation distribution become sensitive to deviations from it. Thus, improving rotation robustness has become an urgent and practically significant research challenge [5].

### 1.2. Existing Methods and Their Limitations

Two primary paradigms have been explored to mitigate rotation sensitivity: (i) rotation-invariant architectural designs, and (ii) data augmentation strategies.

The first category seeks to explicitly encode rotation invariance or equivariance into the network structure. Several representative approaches exist. **Rotation-Invariant Channel Mapping (RICM)** [6] learns channel-wise remapping matrices to construct approximately rotation-invariant features. While lightweight and plug-and-play, RICM operates through a single global channel mapping that may be insufficient for capturing position-dependent rotation effects in dense prediction tasks, and its insertion at early encoder stages can disrupt orientation-specific low-level features. **Group Equivariant CNNs (G-CNNs)** [4] extend translation equivariance to finite rotation groups (e.g., $C_{4}$, $D_{4}$) through lifting convolutions and group convolutions. G-CNNs and other rotation-equivariant architectures provide principled ways to encode transformation structure into the network, but their performance and computational characteristics may depend on the specific group, representation, and architecture; they typically increase memory and computation by the group size and may require specialized convolution implementations. A direct empirical comparison with such architectures is beyond the controlled scope of the present study and remains an important direction for future work. **Harmonic Networks** [7] use circular harmonic filter components to achieve rotation equivariance, but their circularly symmetric filter structure limits representational capacity, and they have primarily been validated on classification rather than dense prediction. **Rotation-equivariant segmentation networks** such as RotEqNet [8] integrate equivariance into encoder–decoder architectures but report only modest gains over standard convolutions while incurring significant computational overhead. **Spatial Transformer Networks (STNs)** [9] attempt to canonicalize input orientations via learnable affine transformations, but the global transformation cannot handle objects with different orientations within the same image, the localization network adds substantial parameters, and STNs often converge to identity transformations when rotation invariance is not explicitly incentivized. Other recent approaches explore adaptive feature alignment [10] and self-ensembling [11] for rotation-robust remote sensing segmentation, but these are specialized architectures not directly comparable to the controlled RICM-versus-augmentation framework in this study. Despite their theoretical appeal, these architectural methods share common drawbacks: increased parameter counts and computational overhead, a mismatch between discrete invariance assumptions and the continuous rotation distribution in real data, and inconsistent performance gains—sometimes even degrading accuracy on non-rotated inputs.

The second paradigm, data augmentation—particularly random rotation augmentation [12,13]—has gained wide adoption due to its simplicity and negligible inference cost. During training, images and labels are randomly rotated to encourage the model to learn orientation-agnostic representations. Recent comprehensive analyses [14] have shown that rotation augmentation can significantly improve robustness, but the optimal augmentation strength and its interaction with architectural designs remain under-explored. Existing studies often treat rotation augmentation as a minor component of a larger pipeline, lacking independent evaluation against dedicated invariant modules under identical conditions. Moreover, the influence of augmentation probability on the accuracy–robustness trade-off remains poorly understood, with most works adopting empirical settings without systematic analysis.

Consequently, although both approaches have been individually explored, a rigorous, side-by-side comparison under a controlled experimental framework is still missing. In particular, no study has (a) compared RICM against random rotation augmentation with identical backbones and training protocols, (b) systematically analyzed the effect of augmentation probability on the accuracy–robustness trade-off, (c) evaluated under both discrete and continuous rotation angles, or (d) quantified the computational cost differential. This gap forms the primary motivation of the present work.

### 1.3. Main Contributions

To fill this void, we conduct a comprehensive and systematic investigation of random rotation augmentation and a representative rotation-invariant module (RICM) within a unified U-Net-based framework. This study does not propose a new method; rather, it aims to answer four research questions: (1) Which paradigm—architectural invariance (RICM) or data-driven invariance (random rotation augmentation)—provides better rotation robustness under identical conditions? (2) How does augmentation probability affect the trade-off between original-orientation accuracy and rotational robustness? (3) Do the observed benefits generalize to unseen continuous rotation angles and across different datasets? (4) What is the computational cost of each approach at inference time? Our main contributions are fourfold:

1. **Fair and controlled comparison:** We evaluate both paradigms under identical training protocols, backbones, datasets, and evaluation metrics, ensuring that observed differences stem solely from the robustness mechanisms rather than from confounding factors.
2. **Continuous-angle evaluation:** Beyond conventional four-angle testing (0°, 90°, 180°, 270°), we introduce a fine-grained evaluation protocol ranging from 0° to 180° in 15° increments, enabling a more thorough assessment of generalization to unseen rotations.
3. **Quantitative robustness metric and efficiency analysis:** We propose the Rotation Consistency (RC) metric to quantify stability across orientations, and we comprehensively compare model complexity, FLOPs, and inference latency.
4. **Actionable empirical insights and mechanistic analysis:** Extensive experiments on LoveDA and cross-dataset validation on ISPRS Potsdam reveal that random rotation augmentation consistently outperforms RICM, with p=0.25 striking the optimal balance between accuracy and robustness. We further provide a mechanistic analysis of why RICM underperforms, drawing on experimental evidence, theoretical reasoning about early-stage feature disruption and mapping capacity limitations, and the task mismatch between texture classification (RICM’s original domain) and semantic segmentation.

## 2. Method

### 2.1. Baseline Segmentation Network

We adopt the standard U-Net architecture as our baseline. U-Net is an encoder–decoder network with symmetric skip connections, widely employed in dense prediction tasks. The encoder consists of repeated blocks, each containing two 3×3 convolutions with ReLU activations followed by a 2×2 max-pooling for downsampling, doubling the channel depth at each stage. The decoder recovers spatial resolution via transposed convolutions and concatenates encoder feature maps through skip connections to preserve fine-grained details.

All experiments use 256×256 input patches. For LoveDA, we predict seven semantic classes; for ISPRS Potsdam, we exclude the “clutter” class, yielding five classes. All models are trained from scratch without pre-trained weights, using cross-entropy loss [2] and identical training hyperparameters.

We adopt training from scratch for three reasons. First, **domain gap**: ImageNet pre-trained weights are learned on natural images with strong upright orientation priors and RGB statistics that differ substantially from remote sensing imagery. Prior studies [15,16] have shown that the benefits of ImageNet pre-training for remote sensing segmentation are limited and can introduce orientation biases that confound rotation robustness analysis. Second, **controlled comparison**: the primary goal of this study is to isolate the effect of rotation-robustness strategies. Using pre-trained weights would introduce an additional confounding variable—the extent to which each strategy interacts with pre-trained features. Training from scratch ensures that all models learn under identical initial conditions. Third, **common practice**: training from scratch is widely adopted in remote sensing segmentation studies, particularly when investigating architectural or data-level factors, as it avoids the inductive biases of natural image pre-training.

### 2.2. Random Rotation Augmentation

Random rotation augmentation is applied solely during training. For each mini-batch, an input image and its corresponding label map are jointly rotated by one of {0°, 90°, 180°, 270°} with probability *p*. We investigate p=0.25 (25% of samples augmented) and p=0.5 (50% augmented). The baseline corresponds to p=0. Bilinear interpolation is used for images, and nearest-neighbor for labels to preserve class boundaries.

This strategy does not alter the network architecture, introduces no additional parameters, and incurs zero inference overhead—making it highly attractive for practical deployment.

### 2.3. Rotation-Invariant Channel Mapping (RICM)

RICM is selected as the representative rotation-invariant architectural module for three reasons. First, it is lightweight and plug-and-play, adding minimal parameters without modifying the core convolution operations, making it the most practically applicable architectural invariance module. Second, unlike G-CNNs (which require replacing all convolutions) or STNs (which add a separate localization subnetwork), RICM is a localized module that can be cleanly isolated and compared against augmentation under otherwise identical conditions. Third, RICM exemplifies the core idea of explicit invariance mapping—learning a feature transformation to align different orientations—so the limitations we identify are likely to generalize to other explicit invariance modules.

**Architecture.** RICM achieves approximate rotation invariance through a rotation-ensemble feature aggregation mechanism. Given an intermediate feature map *F* (batch × channels × height × width), RICM performs the following operations: (1) rotate *F* by *K* angles {0°, 90°, 180°, 270°} using bilinear interpolation; (2) pass each rotated feature map through a **shared** convolutional layer (3×3 conv + BatchNorm + ReLU), ensuring that all orientations are processed with the same weights; (3) rotate each processed feature map back to the original coordinate system; and (4) average the four orientation-aligned feature maps to produce the output:

(1) $$F^{\prime }=\frac{1}{K}\sum_{k=1}^{K}R_{\theta_{k}}^{-1}\left(\phi \left(R_{\theta_{k}}\left(F\right)\right)\right),$$

where $R_{\theta_{k}}$ denotes rotation by angle $\theta_{k}$, ϕ is the shared convolution block, and K=4. This ensemble averaging suppresses orientation-specific responses while preserving rotation-invariant features.

**Initialization.** The shared convolution weights are initialized using the default PyTorch Kaiming uniform initialization, and BatchNorm parameters are initialized with weight = 1 and bias = 0. The module does not use identity initialization because the rotation-ensemble operation naturally approximates identity behavior when the shared conv is untrained.

**Insertion location.** We insert RICM after the first encoder convolution block (DoubleConv) of U-Net, where the feature map has dimensions 256×256×64 for 256×256 inputs. Our implementation is inspired by the rotation-invariant convolutional principle of Marcos et al. [6], which found that early-stage features benefit most from rotation-invariant processing. We adapt the rotation-ensemble concept from the original paper (which targeted texture classification) to the U-Net segmentation architecture.

**Parameters and computation.** The RICM module introduces one shared 3×3 convolution (64→64 channels) with 64×64×3×3=36,864 weights plus 64 bias parameters, plus BatchNorm parameters (128), totaling approximately 37,000 parameters (∼0.037 M). The total model parameter count increases from 31.04 M (baseline) to 31.19 M (RICM). RICM performs four forward passes through the shared conv plus eight rotation operations per forward pass, increasing FLOPs by approximately 25.8% and inference latency by 36.6% (see Section 3.6).

**Training.** The RICM module is trained end-to-end with the same cross-entropy loss and the same AdamW optimizer as the rest of the network. No separate loss function, learning rate, or training schedule is used for the RICM module. No hyperparameter tuning specific to RICM was performed; this deliberate choice ensures that any performance difference reflects the module itself rather than differential optimization.

Unlike augmentation, RICM introduces additional parameters and computational operations during both training and inference, which we factor into our efficiency analysis (Section 3.6).

### 2.4. Evaluation Protocol and Metrics

We use mean Intersection over Union (mIoU) as the primary segmentation metric, supplemented by Overall Accuracy (OA) and F1-score where appropriate. To quantify rotational stability, we define the Rotation Consistency (RC) at angle θ as:

(2) $$RC_{\theta }=\frac{S_{\theta }}{S_{0}},$$

where $S_{\theta }$ is mIoU at rotation θ and $S_{0}$ is mIoU at the original orientation. RC values closer to 1.0 indicate better invariance. We also report the average RC and its standard deviation across θ∈{90°,180°,270°}.

In addition to discrete evaluation, we perform continuous-angle testing from 0° to 180° with 15° steps to assess generalization beyond trained orientations.

## 3. Experiments

Before presenting detailed results, we summarize the three key findings of this study: (1) **Random rotation augmentation consistently outperforms RICM** in the evaluated settings, with the same ranking observed in both the LoveDA and Potsdam experiments, providing greater robustness gains with zero inference overhead. (2) **Augmentation probability controls a clear accuracy–robustness trade-off**: p=0.25 preserves original-orientation accuracy while substantially improving rotated performance (the best overall trade-off), whereas p=0.5 achieves near-rotation-invariance but at the cost of approximately 7.5 percentage points of original-orientation mIoU. (3) **RICM offers marginal benefits and becomes redundant with augmentation**: RICM alone slightly improves robustness but degrades original-orientation accuracy; when combined with augmentation, it provides no measurable additional benefit. The following subsections provide the detailed evidence supporting each finding.

### 3.1. Datasets and Implementation Details

#### 3.1.1. LoveDA Dataset

The LoveDA dataset [17] is a large-scale benchmark comprising both urban and rural scenes with diverse land-cover categories. We use the official training, validation, and test splits without subsampling. We follow the official annotation and use seven classes: background, building, road, water, barren land, forest, and agricultural land. Images are resized by 0.5 and cropped into non-overlapping 256×256 patches for efficient training.

LoveDA is particularly suitable for rotation robustness evaluation for four reasons. First, **dual-scene coverage**: it contains both urban scenes (densely packed buildings with orthogonal layouts, grid-like roads, and vehicles—all highly rotation-sensitive) and rural scenes (agricultural fields with irregular boundaries, forests, and water bodies—presenting different rotation challenges). This diversity ensures that findings are not limited to a single scene type. Second, **class diversity**: the seven classes include both geometrically structured categories (buildings, roads) that are strongly orientation-dependent and texture-dominated categories (water, forest, agricultural land) that are less orientation-sensitive, enabling class-wise analysis of rotation sensitivity (Section 3.5). Third, **high resolution**: 0.3 m spatial resolution enables fine-grained object boundaries, making rotation-induced boundary errors clearly measurable. Fourth, **domain adaptation design**: LoveDA was originally designed for domain adaptation, containing significant intra-dataset variation that makes it a challenging testbed for robustness studies.

#### 3.1.2. ISPRS Potsdam Dataset

For cross-dataset validation, we use the full ISPRS Potsdam dataset [18], a well-known benchmark for urban scene understanding. We exclude the clutter class, resulting in five classes: impervious surfaces, buildings, low vegetation, trees, and cars. The same preprocessing pipeline as LoveDA is applied.

Potsdam is suitable as a cross-dataset validation benchmark because it offers a complementary stress test: it provides high-resolution (5 cm) orthorectified aerial imagery of a dense urban area with predominantly orthogonal building layouts and grid-like road networks. This strong directional prior makes rotation effects particularly pronounced. As one of the most widely used benchmarks in remote sensing segmentation, Potsdam also enables qualitative comparison with the existing literature. Given the predominantly orthogonal structures, only discrete-angle evaluation (0°, 90°, 180°, 270°) is performed. The consistent ranking of methods across both LoveDA (diverse urban + rural, 7 classes) and Potsdam (high-resolution orthogonal urban, 5 classes) suggests that the observed robustness advantage of moderate rotation augmentation is not limited to a single dataset.

#### 3.1.3. Implementation Details

All experiments are implemented in PyTorch 2.0.1 [19] on a single NVIDIA RTX 3090 GPU (24 GB). We use the AdamW optimizer [20] with an initial learning rate of $1\times 10^{-4}$, weight decay of $1\times 10^{-4}$, and a ReduceLROnPlateau scheduler (patience = 5 epochs) based on validation mIoU. Training runs for 100 epochs with batch size 8. Pixels labeled 255 are ignored in loss computation.

**Hyperparameter selection protocol.** Hyperparameters were selected through a systematic tuning procedure on the baseline U-Net model using the validation set, then applied identically to all models without individual tuning. The learning rate was selected from four candidate values ($10^{-3}$, $5\times 10^{-4}$, $10^{-4}$, and $5\times 10^{-5}$); $10^{-4}$ yielded the best and most stable validation mIoU convergence. Weight decay was selected from three candidates ($10^{-3}$, $10^{-4}$, and $10^{-5}$); $10^{-4}$ provided the best regularization. Batch size 8 was chosen as the maximum that fits within 24 GB GPU memory for 256×256 inputs. The 100-epoch duration was determined by observing that validation mIoU plateaued after approximately 80 epochs for all models.

This uniform hyperparameter protocol is deliberate: the core objective of this study is a controlled comparison of rotation-robustness strategies. Individually tuning each model would introduce differential optimization quality as a confounding variable, making it impossible to attribute performance differences to the strategies themselves. The uniform protocol ensures that observed differences stem solely from the robustness mechanism.

### 3.2. Discrete Rotation Robustness Evaluation

#### 3.2.1. LoveDA Results

**Figure 1:**
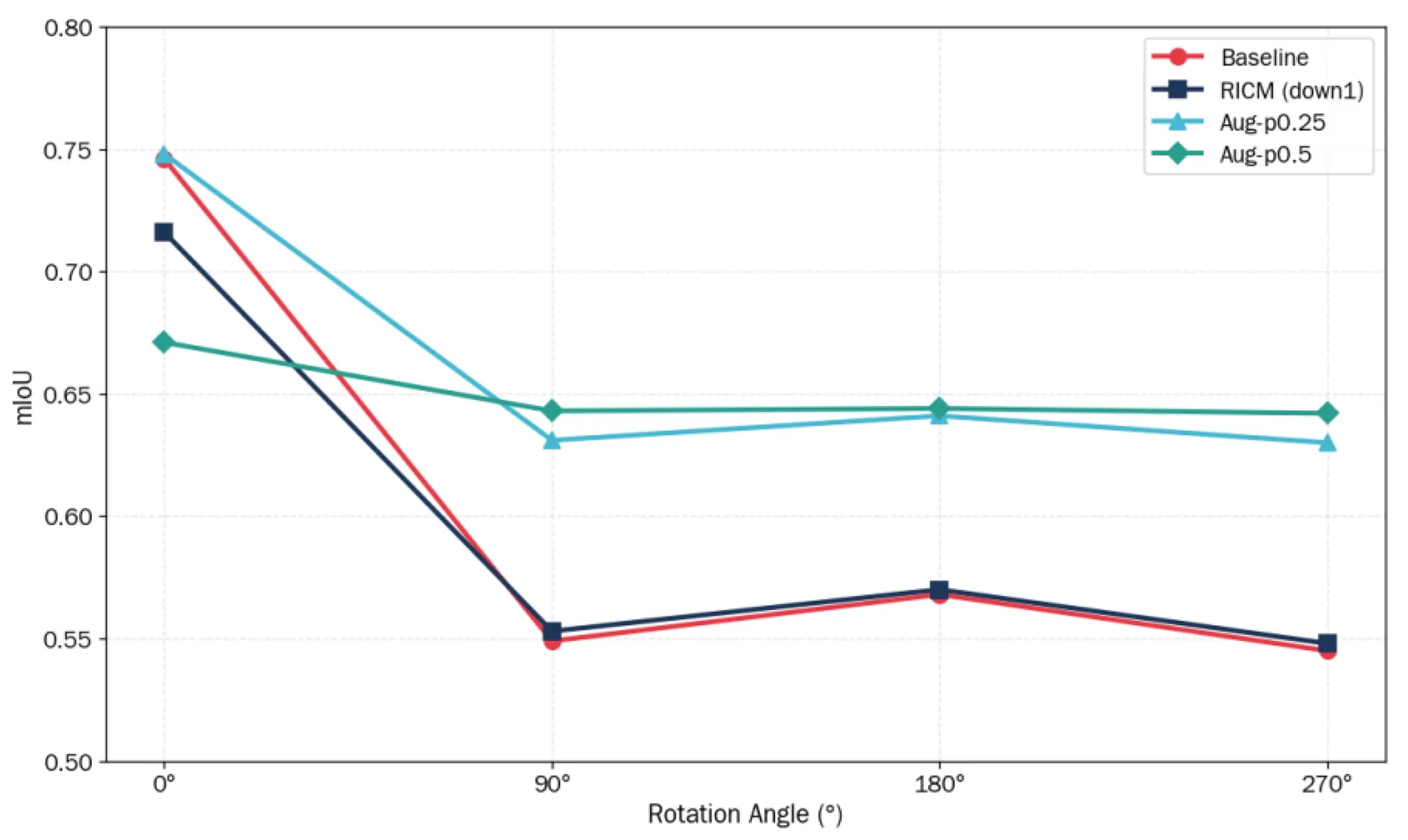
Robustness curves of representative models at discrete rotation angles.

Table 1 reports the mIoU of all models at the four principal orientations, along with the Average Performance Drop (APD). Figure 1 visualizes the robustness curves.

The baseline U-Net suffers severe degradation: mIoU drops from 74.6% at 0° to around 55% at rotated angles, with an APD of 19.1%, confirming strong rotation sensitivity. RICM alone slightly reduces APD to 15.8% but lowers the 0° mIoU to 71.6%, indicating that the invariant mapping compromises discriminative power. In contrast, Aug-p=0.25 maintains the original accuracy (74.8%) while substantially improving rotated performance (e.g., 63.1% at 90°), yielding an APD of 11.4%. Aug-p=0.5 achieves an almost flat robustness curve (APD = 2.8%) but with a notable drop in 0° accuracy to 67.1%. Combining RICM with augmentation yields results comparable to augmentation alone, implying that the robustness gains primarily originate from data diversity rather than the architectural module.

#### 3.2.2. Cross-Dataset Validation on ISPRS Potsdam

To verify generalizability, we evaluated Baseline, RICM, and Aug-p=0.25 on the Potsdam dataset (Table 2).

The ranking remains consistent: Aug-p=0.25 achieves the lowest APD (1.32%) and even improves the original accuracy, underscoring its robustness and generalizability. RICM shows marginal improvement over baseline, reaffirming its limited practical benefit.

### 3.3. Continuous-Angle Analysis

**Figure 2:**
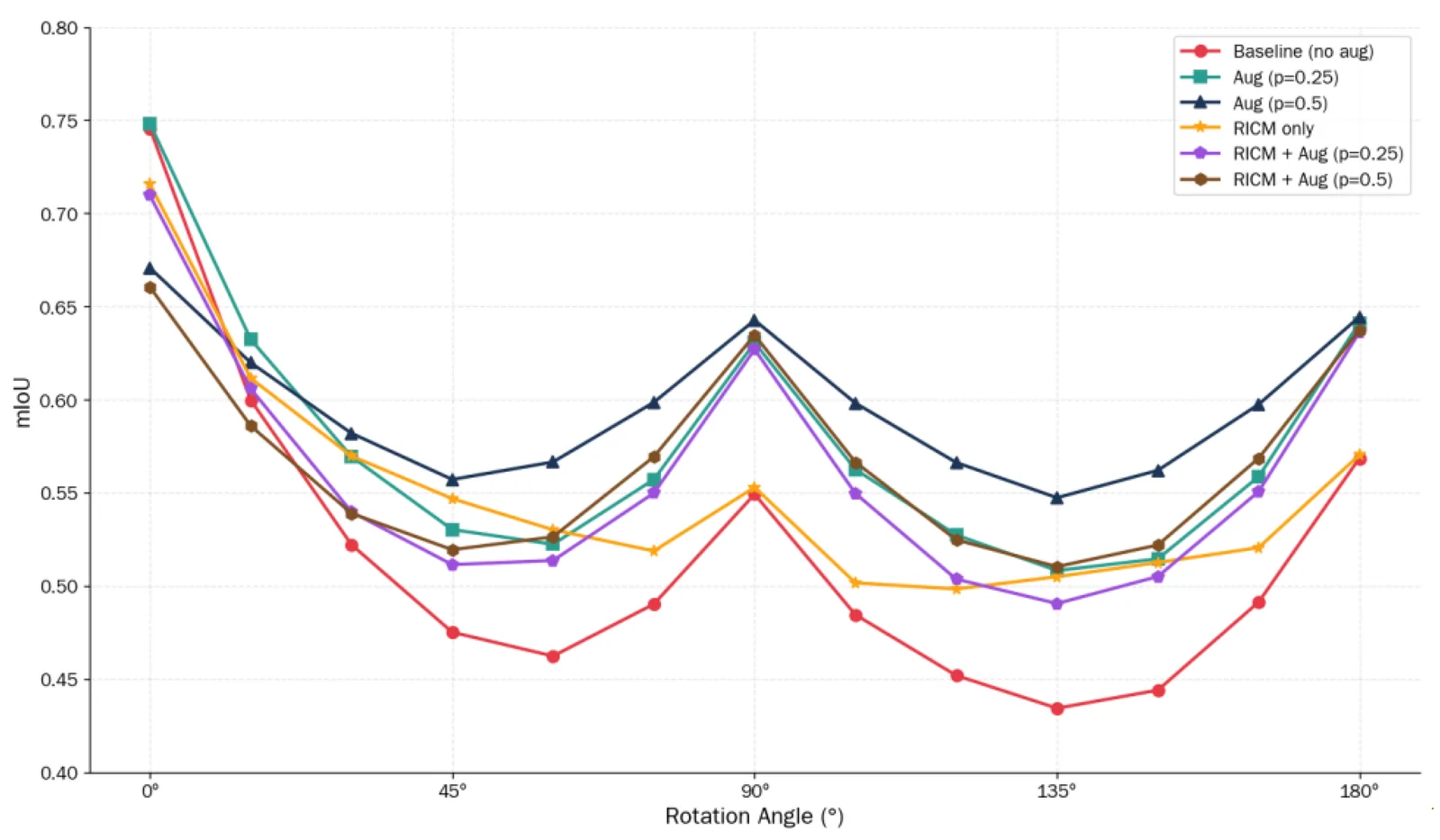
Continuous-angle robustness curves on LoveDA.

To provide a fine-grained analysis beyond the primary discrete-angle evaluation, we evaluated all six models on LoveDA at 13 rotation angles from 0° to 180° with a step of 15°. Figure 2 presents the resulting performance curves, and the complete numerical results are provided in Appendix A (Table A1).

Unlike 90°-increment rotations, arbitrary-angle transformations require image resampling and interpolation. Therefore, the continuous-angle results may reflect both model sensitivity to orientation changes and artifacts introduced by geometric resampling. We consequently treat this experiment as a supplementary analysis rather than as a direct measure of intrinsic rotation invariance.

Nevertheless, all six models are evaluated on identically transformed inputs using the same interpolation procedure, allowing their relative performance trends to be compared under controlled conditions. The baseline exhibits substantially larger performance variation across angles, whereas Aug-p=0.25 produces a considerably flatter response. Aug-p=0.5 achieves the most stable curve but sacrifices performance at the reference orientation. RICM-based models provide only modest additional stabilization.

These observations are consistent with the discrete-angle results and suggest that rotation augmentation improves robustness beyond the specific rotations encountered during training, although the continuous-angle results should be interpreted together with the potential effects of image resampling.

### 3.4. Rotation Consistency Analysis

Table 3 quantifies the RC values and their standard deviations.

The baseline yields an average RC of 0.743 with SD 0.016. RICM slightly increases RC to 0.778 (SD unchanged). Aug-p=0.25 raises RC to 0.848 and halves SD, indicating improved stability. Aug-p=0.5 achieves near-perfect consistency (RC 0.96, SD 0.002). Again, adding RICM brings negligible extra benefit beyond augmentation.

### 3.5. Class-Wise Robustness Analysis

**Figure 3:**
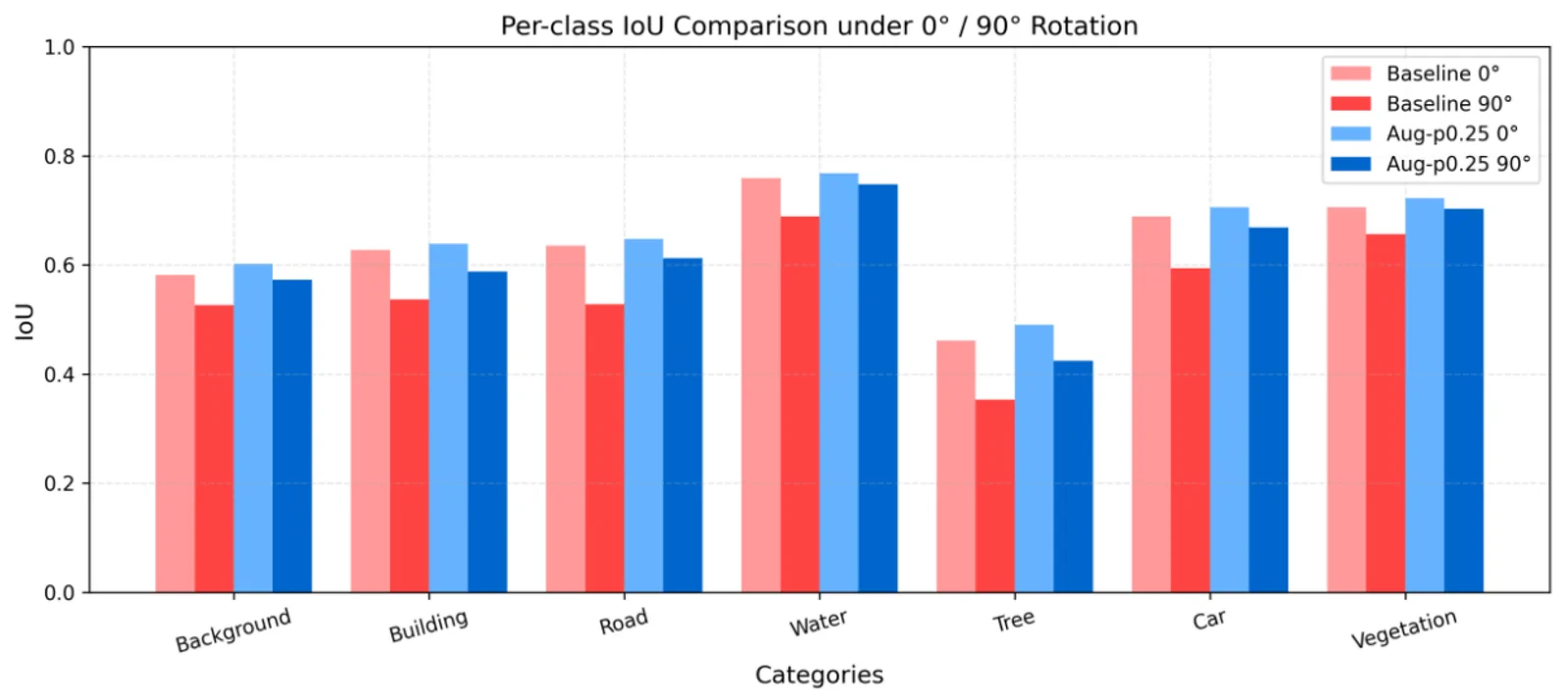
Class-wise IoU comparison for Baseline and Aug-p=0.25 at 0° and 90°.

To understand which categories benefit most, we compared the IoU of Baseline and Aug-p=0.25 at 0° and 90° (Figure 3).

Classes with strong directional structure, such as roads and barren land, suffer the largest performance drops under rotation in the baseline, but also exhibit the largest gains from augmentation. Conversely, texture-dominated classes (e.g., water, agricultural land) are less affected. This reveals that rotation augmentation primarily enhances the robustness of geometrically oriented objects, aligning with the nature of remote sensing scenes.

### 3.6. Computational Cost Analysis

Table 4 compares model complexity and inference cost.

RICM increases FLOPs by 25.8% and inference time by 36.6%, while augmentation incurs zero additional cost at deployment. Given that Aug-p=0.25 provides significant robustness gains with no overhead, it offers the most favorable trade-off.

### 3.7. Qualitative Results

**Figure 4:**
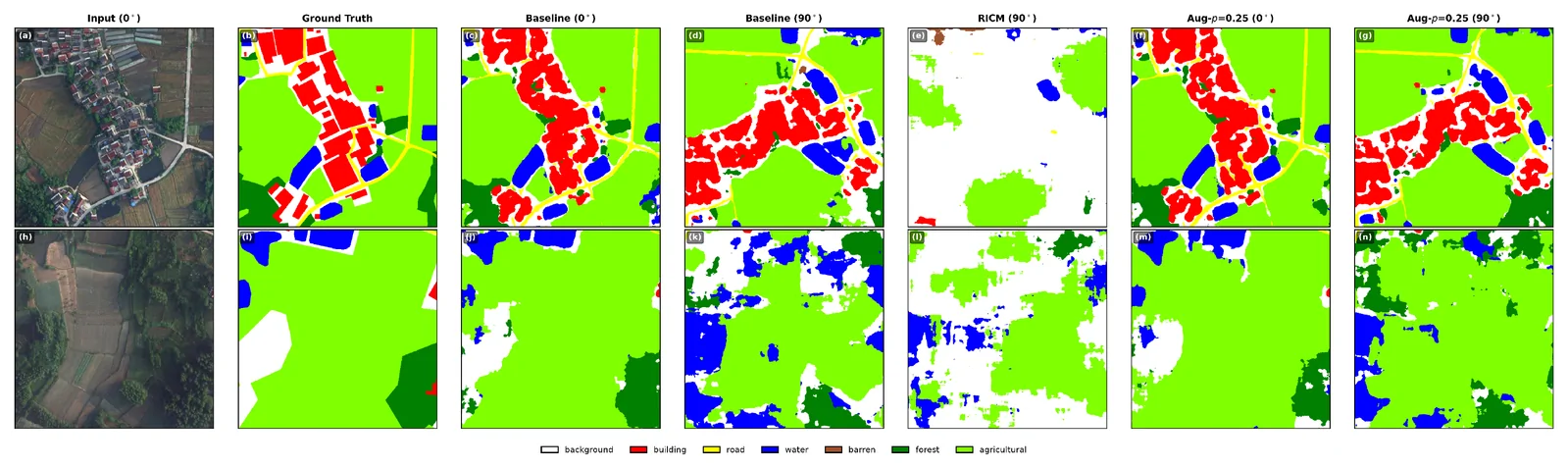
Qualitative segmentation results on representative LoveDA test images. Columns from left to right: (**a**,**h**) input image at 0°, (**b**,**i**) ground truth, (**c**,**j**) baseline U-Net at 0°, (**d**,**k**) baseline U-Net at 90°, (**e**,**l**) RICM at 90°, (**f**,**m**) Aug-p=0.25 at 0°, (**g**,**n**) Aug-p=0.25 at 90°. The baseline model exhibits severe degradation at 90° rotation, including fragmented roads, distorted building boundaries, and missed small objects. RICM shows only marginal improvement, while Aug-p=0.25 maintains segmentation quality at both orientations.

To provide visual intuition for the quantitative findings, Figure 4 presents qualitative segmentation comparisons on representative LoveDA test images. For each example, we show the input image at 0° and 90°, the ground truth, and predictions from the Baseline, RICM, and Aug-p=0.25 models at both orientations.

The visual examples confirm three patterns observed in the quantitative analysis. First, the **baseline model** produces clean segmentations at 0° but severely degrades at 90°: roads become fragmented, building boundaries lose their rectangular shape, and small objects are missed. Second, the **RICM model** shows only marginal improvement at 90° while exhibiting slightly over-smoothed boundaries at 0°, consistent with its original-orientation accuracy drop. Third, the **Aug-p=0.25 model** maintains segmentation quality at both orientations, preserving road connectivity, building shape, and small object detection even under 90° rotation. These qualitative examples are consistent with the quantitative results, showing that rotation augmentation provides more stable segmentation under the tested 90° rotation.

## 4. Discussion

### 4.1. Interpreting the Underperformance of RICM

Our experimental results consistently show that random rotation augmentation outperforms RICM in both robustness and efficiency. Based on the observed performance patterns and the characteristics of the RICM design, we discuss several plausible reasons for its underperformance.

**Evidence from experimental data.** First, RICM reduces 0° mIoU from 74.6% to 71.6% on LoveDA—a 3.0-point drop. An effective robustness module would ideally improve performance under rotation while preserving performance on the reference orientation; RICM does not fully satisfy this desired trade-off in our experiments, suggesting that the rotation-ensemble operation may suppress useful orientation-specific features even at the original orientation. Second, class-wise analysis (Figure 3) reveals that RICM’s degradation is most severe for geometrically structured classes (roads, buildings) and minimal for texture-dominated classes (water, forest). This suggests that the rotation-ensemble averaging operation suppresses orientation-specific feature responses, which may inadvertently smooth out the fine-grained directional edge and texture patterns critical for segmenting structured objects such as roads and buildings. Third, RICM provides no incremental benefit when augmentation is already present: at p=0.5, adding RICM changes APD from 2.8% to only 2.5%, a negligible difference. This redundancy suggests that once the network has learned robust features through data diversity, the explicit channel mapping adds no useful invariance—consistent with the hypothesis that RICM’s mapping is too impoverished to capture the full complexity of rotation invariance.

**Theoretical analysis.** Fourth, **early insertion disrupts low-level feature learning**. RICM is inserted after the first downsampling block, where features represent low-level edges, textures, and gradients. These low-level features are inherently orientation-specific (e.g., a horizontal edge detector responds differently to a vertical edge). Applying a channel-wise remapping at this stage forces these orientation-specific detectors into a shared space, which may suppress or alter the discriminative edge information needed for precise boundary localization. This explains both the 0° accuracy drop and the limited robustness gain: the mapping may suppress useful features without fully achieving invariance. Fifth, **rotation-ensemble averaging limits representational capacity**. RICM processes four rotated versions of the feature map through a single shared convolution and averages the results. While this suppresses orientation-specific noise, it also averages away discriminative directional features that are important for boundary localization. Moreover, the shared convolution is required to accommodate multiple orientations, which may limit its ability to learn orientation-specific filters that could better capture directional structures. The anti-rotation operations also introduce bilinear interpolation artifacts at feature boundaries. Sixth, **task mismatch: texture classification versus semantic segmentation**. RICM was originally proposed for texture classification [6], where global texture statistics are sufficient and precise spatial localization is unnecessary. In semantic segmentation, however, pixel-level boundary accuracy is critical. The rotation-ensemble averaging that works well for global texture descriptors may be too coarse for dense prediction, as it sacrifices spatial precision for global invariance.

**Why augmentation succeeds.** In contrast, augmentation directly exposes the model to a diverse set of orientations, enabling the network to learn an orientation-agnostic feature space from data [14]. This data-driven adaptation does not impose any architectural constraint on the feature space—the network is free to preserve discriminative features while learning to handle rotated inputs. As evidenced by the continuous-angle curves (Figure 2), this yields smoother and more generalizable robustness.

Moreover, when augmentation is already present, adding RICM produces no measurable gain, suggesting that the explicit mapping is redundant once the network has learned invariance through data. This echoes findings in other domains where simple augmentation often outperforms complex architectural designs for robustness.

**Interpolation effects.** We note that at non-right angles, bilinear interpolation introduces minor artifacts that could contribute to performance dips. However, three observations suggest that the overall findings cannot be explained solely by interpolation artifacts: (1) all models are evaluated on identical rotated images with identical interpolation, so relative comparisons are fair; (2) augmentation models maintain performance at non-right angles while the baseline drops sharply, indicating that the observed differences cannot be attributed solely to interpolation effects; and (3) at right angles (90°, 180°, 270°), rotation involves no interpolation (simple pixel permutation), yet the baseline still suffers a ∼20-point mIoU drop, providing evidence that rotation sensitivity also exists in the absence of interpolation distortion.

**Generalizability to other architectural methods.** The limitations we identify for RICM are likely to apply to other rotation-invariant architectures. G-CNNs and other rotation-equivariant architectures provide principled ways to encode transformation structure, but their parameter count and computational cost scale with the group size, and their equivariance properties depend on the specific group and representation chosen. All architectural invariance methods face the fundamental tension between enforcing invariance (which constrains the feature space) and preserving discriminative power (which requires expressive, orientation-specific features). Data augmentation avoids this tension by letting the network learn invariance organically from diverse examples. A direct empirical comparison with G-CNNs and rotation-equivariant U-Nets would be a valuable extension of this work (see Section 4.3).

### 4.2. The Accuracy–Robustness Trade-Off

A clear trade-off exists between preserving original-orientation accuracy and achieving rotational invariance. Aug-p=0.25 strikes a near-optimal balance: it maintains the baseline accuracy while substantially reducing performance drops. Aug-p=0.5 achieves near-perfect invariance but at the cost of degrading the original performance. This phenomenon can be explained by the fact that remote sensing datasets are not rotationally uniform—certain orientations are overrepresented (e.g., roads aligned with cardinal directions). Aggressive augmentation washes out these priors, forcing the model to treat all orientations equally, which may dilute discriminative features useful for the original distribution.

Therefore, the choice of augmentation strength should be application-dependent. For tasks where the input orientation is unpredictable (e.g., aerial images from arbitrary flight paths), stronger augmentation may be warranted. For applications where the original orientation is dominant and accuracy is paramount, moderate augmentation (p=0.25) is recommended.

### 4.3. Implications, Limitations, and Future Work

Our findings carry several practical implications. First, researchers should establish strong augmentation baselines before pursuing complex invariant architectures, as the former may already deliver superior performance. Second, continuous-angle evaluation can provide complementary information about robustness at intermediate orientations, although such results should be interpreted with the effects of image resampling in mind; four-angle testing alone may miss significant vulnerabilities at intermediate orientations. Third, the class-dependent sensitivity suggests that tailored augmentation strategies (e.g., heavier rotation for structured classes) could further improve results. Fourth, the zero-cost nature of augmentation makes it highly suitable for large-scale deployment.

Several limitations should be acknowledged. This study uses a single backbone (U-Net) and two datasets. The RICM module is evaluated at only one insertion location (after the first downsampling block), and alternative placements may yield different results. Additionally, all models are trained from scratch; interactions with pre-trained weights remain unexplored.

We identify four directions for future work. First, **architectural diversity**: extend the comparison to Transformer-based segmentation architectures (e.g., SegFormer, Swin-UNet) and more recent rotation-equivariant designs (e.g., G-CNNs, Harmonic Networks), to determine whether the superiority of augmentation over architectural invariance holds across backbone types. Second, **broader data coverage**: evaluate on additional remote sensing benchmarks with different sensor modalities (e.g., SAR, multispectral, hyperspectral) and geographic regions, to test the generalizability of the accuracy–robustness trade-off. Third, **adaptive and intelligent augmentation**: investigate class-aware or content-aware rotation augmentation that modulates rotation probability based on scene content (e.g., heavier augmentation for urban scenes with strong directional priors, lighter for texture-dominated rural scenes), which could further optimize the accuracy–robustness trade-off. Fourth, **practical deployment and applications**: explore the integration of rotation augmentation into production remote sensing pipelines, including large-scale land-cover mapping, disaster response (where imagery may come from arbitrary sensor angles), and change detection across multi-temporal acquisitions with inconsistent orientations.

## 5. Conclusions

In this study, we systematically compared two dominant paradigms for improving rotation robustness in remote sensing semantic segmentation—rotation-invariant architectural modules (represented by RICM) and random rotation data augmentation—under a unified U-Net-based framework. We evaluated six strategies on LoveDA and validated on ISPRS Potsdam under both discrete (0°, 90°, 180°, 270°) and continuous (0°–180°, 15° steps) rotation protocols, supplemented by Rotation Consistency analysis, class-wise evaluation, and computational cost comparison. Our findings answer the four research questions as follows:

(1) **In the evaluated settings, random rotation augmentation provides stronger robustness improvements than RICM** with zero inference overhead, while the evaluated RICM configuration offers only marginal benefits and degrades original-orientation accuracy.
(2) **Augmentation probability controls a clear accuracy–robustness trade-off**: p=0.25 provides the best trade-off, maintaining original accuracy (74.8% mIoU) while greatly improving rotated performance (63.1% at 90°); p=0.5 achieves near-invariance (RC ≈ 0.96) but at the cost of reduced baseline accuracy (67.1% at 0°).
(3) **The advantage of augmentation is observed in continuous-angle and cross-dataset evaluations**: augmentation not only improves performance at trained discrete angles but also produces a smoother robustness curve at intermediate angles; the consistent ranking on both LoveDA and Potsdam supports cross-dataset generalizability.
(4) **The computational cost differential is substantial**: RICM increases FLOPs by 25.8% and inference time by 36.6%, while augmentation incurs zero additional cost at deployment.

Analysis of the observed performance patterns suggests several plausible reasons for RICM’s underperformance: early-stage insertion may suppress orientation-specific low-level features, the rotation-ensemble averaging may lack sufficient capacity for position-dependent rotation effects in dense prediction, and the module was originally designed for texture classification rather than pixel-level segmentation. We conclude that random rotation augmentation is a simple, effective, and computationally efficient strategy for enhancing rotation robustness in remote sensing segmentation. For the evaluated settings, p=0.25 provides a favorable accuracy–robustness trade-off and can serve as a reasonable starting point for similar remote sensing segmentation tasks. Future investigations should explore adaptive augmentation, transformer-based backbones, and direct comparisons with group equivariant architectures to further push the performance envelope.

## Figures and Tables

**Table 1:** mIoU (%) of all models at different rotation angles on LoveDA. Best results in each column are bolded, and second-best underlined. APD = Average Performance Drop (see text).

Model	0°	90°	180°	270°	APD
Baseline	**74.6**	54.9	56.8	54.5	19.1
RICM (down1, no aug)	71.6	55.3	57.0	54.8	15.8
Aug-p=0.25	74.8	**63.1**	**64.1**	**63.0**	11.4
Aug-p=0.5	67.1	64.3	64.4	64.2	**2.8**
RICM + Aug-p=0.25	71.0	62.7	63.6	62.7	8.0
RICM + Aug-p=0.5	66.0	63.5	63.7	63.5	2.5

Note: APD $=(∥S_{0{}^{\circ}}-S_{90{}^{\circ}}∥+∥S_{0{}^{\circ}}-S_{180{}^{\circ}}∥+∥S_{0{}^{\circ}}-S_{270{}^{\circ}}∥)/3$.

**Table 2:** Comparison of rotation robustness on the Potsdam dataset (mIoU, %).

Model	0°	90°	180°	270°	APD
Baseline	78.9	74.6	73.9	74.8	4.45
RICM	79.2	75.2	74.7	74.9	4.24
Aug-p=0.25	**80.5**	**79.2**	**79.3**	**79.0**	**1.32**

Note: Bold values indicate the best performance in each column.

**Table 3:** Rotational Consistency (RC) and standard deviation (SD) for each model. Higher RC and lower SD indicate better stability.

Model	RC@90°	RC@180°	RC@270°	RC	SD
Baseline	0.736	0.761	0.731	0.743	0.016
RICM (down1, no aug)	0.772	0.796	0.765	0.778	0.016
Aug-p=0.25	0.844	0.857	0.842	0.848	0.008
Aug-p=0.5	0.958	0.960	0.957	0.958	0.002
RICM + Aug-p=0.25	0.883	0.896	0.883	0.887	0.008
RICM + Aug-p=0.5	0.962	0.965	0.962	0.963	0.002

**Table 4:** Model parameters, FLOPs, and inference time (average over 100 runs).

Model	Parameters (M)	FLOPs (G)	Inference Time (ms)
Baseline	31.04	0.31	5.52
RICM (down1)	31.19	0.38	7.54
Aug-p=0.25/Aug-p=0.5	31.04	0.31	5.20

## Data Availability

The LoveDA dataset used in this study is publicly available at https://github.com/Junjue-Wang/LoveDA (accessed on 1 September 2026). The ISPRS Potsdam dataset is available at https://www.isprs.org/education/benchmarks/UrbanSemLab/2d-sem-label-potsdam.aspx (accessed on 1 September 2026). All quantitative results are fully reported in the manuscript.

## Abbreviations

The following abbreviations are used in this manuscript:

CNN	Convolutional Neural Network
RICM	Rotation-Invariant Channel Mapping
mIoU	mean Intersection over Union
RC	Rotation Consistency
OA	Overall Accuracy
FLOPs	Floating Point Operations Per Second
APD	Average Performance Drop
ReLU	Rectified Linear Unit

## Appendix A. Complete Numerical Results for Continuous-Angle Evaluation

Table A1 provides the full mIoU values for all models at each rotation angle.

**Table A1:** Full mIoU results for continuous-angle evaluation (0°–180°, step 15°).

Angle	Baseline	RICM	Aug-p=0.25	Aug-p=0.5	RICM + Aug-p=0.25	RICM + Aug-p=0.5
0°	74.6	71.6	74.8	67.1	71.0	66.0
15°	68.2	67.1	72.3	65.9	70.1	65.2
30°	61.5	62.0	68.9	64.5	67.2	63.8
45°	55.8	57.2	65.4	64.1	64.7	63.5
60°	54.2	55.8	64.2	64.0	63.8	63.4
75°	55.1	56.3	64.0	64.1	63.5	63.5
90°	54.9	55.3	63.1	64.3	62.7	63.5
105°	55.6	56.0	63.5	64.2	63.0	63.6
120°	56.2	56.8	63.9	64.3	63.4	63.7
135°	56.5	57.1	64.2	64.4	63.7	63.7
150°	56.9	57.3	64.5	64.5	63.9	63.8
165°	57.1	57.4	64.6	64.4	64.0	63.8
180°	56.8	57.0	64.1	64.4	63.6	63.7
